# Isolation of syncytiotrophoblast microvesicles and exosomes and their characterisation by multicolour flow cytometry and fluorescence Nanoparticle Tracking Analysis

**DOI:** 10.1016/j.ymeth.2015.03.028

**Published:** 2015-10-01

**Authors:** R.A. Dragovic, G.P. Collett, P. Hole, D.J.P. Ferguson, C.W. Redman, I.L. Sargent, D.S. Tannetta

**Affiliations:** a Nuffield Department of Obstetrics & Gynaecology, University of Oxford, Womens Centre Level 3, John Radcliffe Hospital, Oxford OX3 9DU, UK; b Malvern Instruments, London Road, Minton Park, Amesbury SP4 7RT, UK; c Nuffield Department of Clinical Laboratory Science, University of Oxford, John Radcliffe Hospital, OX3 9DU Oxford, UK

**Keywords:** EV, extracellular vesicle, fl-NTA, fluorescence Nanoparticle Tracking Analysis, FSC, forward scatter, HLA, human leukocyte antigen, LDL, low density lipoprotein, mPerf, maternal side perfusate, NTA, Nanoparticle Tracking Analysis, PLAP, placental alkaline phosphatase, Qdots, quantum dots, RBC, red blood cell, SN, supernatant, SSC, side-scatter, STB, syncytiotrophoblast, STBEX, syncytiotrophoblast exosome, STBEV, syncytiotrophoblast extracellular vesicle, STBMV, syncytiotrophoblast microvesicle, TEM, transmission electron microscopy, VLDL, very low density lipoprotein, Extracellular vesicles, Microvesicles, Exosomes, Flow cytometry, Fluorescence NTA, Placenta perfusion

## Abstract

The human placenta releases multiple types and sizes of syncytiotrophoblast (STB) extracellular vesicles (EV) into the maternal circulation that exhibit diverse biological activities. The placental perfusion technique enables isolation of these STBEV, but conventional flow cytometry can only be used to phenotype EV down to ∼300 nm in size. Fluorescence Nanoparticle Tracking Analysis (fl-NTA) has the potential to phenotype EV down to ∼50 nm, thereby improving current characterisation techniques. The aims of this study were to prepare microvesicle and exosome enriched fractions from human placental perfusate (*n* = 8) and improve fl-NTA STBEV detection. Differential centrifugation and filtration effectively removed contaminating red blood cells from fresh placental perfusates and pelleted a STB microvesicle (STBMV) fraction (10,000×*g* pellet – 10KP; NTA modal size 395 ± 12 nm), enriched for the STB marker placental alkaline phosphatase (PLAP) and a STB exosome (STBEX) fraction (150,000×*g* pellet – 150KP; NTA modal size 147 ± 6 nm), enriched for PLAP and exosome markers Alix and CD63. The PLAP positivity of ‘standard’ 10KP and 150KP pools (four samples/pool), determined by immunobead depletion, was used to optimise fl-NTA camera settings. Individual 10KP and 150KP samples (*n* = 8) were 54.5 ± 5.7% (range 17.8–66.9%) and 30.6 ± 5.6% (range 3.3–51.7%) PLAP positive, respectively. We have developed a reliable method for enriching STBMV and STBEX from placental perfusate. We also standardised fl-NTA settings and improved measurement of PLAP positive EV in STBMV. However, fl-NTA is not as sensitive as anti-PLAP Dynabead capture for STBEX detection, possibly due to STBEX having lower surface expression of PLAP. These important developments will facilitate more detailed studies of the role of STBMV and STBEX in normal and pathological pregnancies.

## Highlights

• Placental perfusion yields large amounts of STBEV.
• Differential centrifugation isolates enriched preparations of STBMV and STBEX.
• fl-NTA can be optimised using an EV ‘standard’.
• fl-NTA reliably detects STBMV using the specific marker PLAP.

## Graphical abstract

**Figure:**
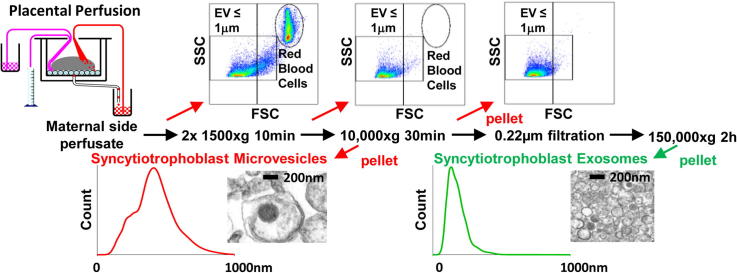

## 1 Introduction

Extracellular vesicles (EV) have been shown to be released by many cell types, both as part of normal physiology and disease processes. These are derived from either membranous extrusions from the plasmalemma (apoptotic blebs (∼1–5 μm)) and microvesicles (∼100 nm–1μm) or multivesicular bodies of the endocytic pathway (exosomes (∼50–200 nm)) that are formed and released by specific mechanisms and as such could contain very different cargoes and have diverse biological effects [1]. Most work has been focussed on the study of exosomes released by cells *in vitro*, however EV release from tissues is far more complex and in reality all forms of EV need to be studied. The syncytiotrophoblast (STB) layer of the human placenta is a highly specialised polarised epithelium covering the entire surface of the placenta, which releases all three vesicle types directly into the maternal bloodstream. These are collectively known as syncytiotrophoblast extracellular vesicles (STBEV). In addition much larger syncytial nuclear aggregates (20–500 μm) are secreted that are unique to the STB [2], [3], [4], [5]. STBEV have been implicated in both maintenance of normal pregnancy and the pathophysiology of disorders of pregnancy, in particular pre-eclampsia [6]. Due to their complex cargo of proteins, lipids and nucleic acids, STBEV constitute a major signalling mechanism between the placenta, a semiallogeneic fetal tissue, and the maternal immune and cardiovascular systems [7].

STBEV have been prepared for research purposes using various *in vitro* and *ex vivo* methods. These include placental explant culture, spontaneous fusion of isolated term placenta primary villous cytotrophoblast to form STB *in vitro*, induced fusion and differentiation of the BeWo trophoblast cell line in culture (G Collett unpublished data) and *ex vivo* dual placental lobe perfusion [7], [8], [9], [10], [11], [12], [13], [14]. There are advantages and disadvantages with each method, such as contamination with EV from cells other than STB when using placental tissue and placenta derived cells; low STBEV yields from placental explant and trophoblast cultures, and disruption of the placental architecture in explant and primary trophoblast cultures with the associated loss of the existing STB layer. This is particularly detrimental when studying pre-eclampsia and the related changes to the STB. Taking these caveats into account, dual placental lobe perfusion was the method chosen to produce STBEV for this study as the placental architecture and most importantly, the STB layer are maintained and a relatively large surface area of the placenta is sampled. As such, the yield of STBEV will be greater and contain EV most representative of the placenta when *in utero*.

The simultaneous release of multiple types of EV further complicates the isolation of STBEV subtypes. EV have been isolated from various biological fluids by differential centrifugation, density gradient centrifugation, liquid chromatography, immunobead capture and filtration methods [15]. Again, each technique has its advantages and disadvantages. For example, liquid chromatography may require pre- and post-run sample processing that extends the overall preparation time and reduces yields, owing to increased handling and inherent losses at each step; methods such as liquid chromatography, density gradient centrifugation and filtration, that have a fixed working range, may only be effective for the isolation of EV within a narrow size distribution such as exosomes; isolation methods based on size, e.g. liquid chromatography and filtration, may not be effective at removing contaminating particles with a size distribution overlapping that of EV, such as lipoproteins (chylomicrons, LDLs and VLDLs) which heavily contaminate samples containing blood, and finally immunobead capture becomes financially restrictive with large sample volumes and numbers. Differential centrifugation was chosen in the current study as it fulfilled the requirements of no pre-centrifugation sample processing, compatibility with the large sample volume produced in the perfusion system (∼400–600 mL) and the ability to separate out microvesicles and exosomes from the same sample at relatively low costs.

Validation of an isolation technique is essential to establish its value. This requires the ability to size, count and phenotype the EV present at each stage of the isolation procedure. This can be achieved using a panel of routine techniques such as transmission electron microscopy (TEM), Nanoparticle Tracking Analysis (NTA), multi-colour flow cytometry and Western blotting, as used in the present study. Multiple techniques are required because no one method can size, count and phenotype EV simultaneously in biological samples that contain a polydispersed population of EV [16]. Fluorescence Nanoparticle Tracking Analysis (fl-NTA) has the potential to phenotype EV down to ∼50 nm in size, which is not possible using conventional flow cytometry [17]. However, unlike flow cytometry, fl-NTA cannot currently measure EV in scatter and fluorescence modes simultaneously. Also, the depth of volume from which EV are counted is dependent on EV brightness set by the camera level and/or the detection threshold used. To ensure that EV measured by scatter and fluorescence are measured from within the same volume, it is necessary to ensure both these measurements are made at a camera level which makes the visible EV approximately the same brightness, which would typically mean a higher camera level for fluorescence measurements. To establish optimal camera levels for each mode, a ‘standard’ containing a known amount of EV positive for the antigen being studied is required. Therefore, we prepared an EV standard to optimise fl-NTA settings to quantify placental alkaline phosphatase (PLAP – a trophoblast marker) positive EV in preparations of STB microvesicles (STBMV) and exosomes (STBEX).

Investigation of the role of STBMV and STBEX in normal and pathological pregnancies is hampered by the low levels circulating in the mother compared to maternal platelet and red blood cell (RBC) EV [4]. Dual placental lobe perfusion enables the preparation of large quantities of STBEV most closely resembling those released *in vivo*, while differential centrifugation allows the sequential isolation of fractions enriched for microvesicles and exosomes from large sample volumes. fl-NTA has the potential to extend our current capabilities and enable phenotyping of nano-sized EV. Therefore, the aims of this study were (1) to develop a robust centrifugation method for isolating highly enriched preparations of STBMV and STBEX from maternal side placental perfusate and (2) to standardise existing fl-NTA methods and improve phenotyping of STBEV.

## 2 Materials and methods

### 2.1 Patient information

Placentas (*n* = 8) were collected within 10 min of delivery from normal pregnant women undergoing elective caesarean sections without labour. Normal pregnancy was defined as a healthy singleton pregnancy with no history of chronic illness and no hypertension or proteinuria during this pregnancy. The Oxfordshire Research Ethics Committee C approved this study (Ref. 09/H0606/10) and informed written consent was obtained from all recruits.

### 2.2 Dual placental lobe perfusion model

**Fig. 1:**
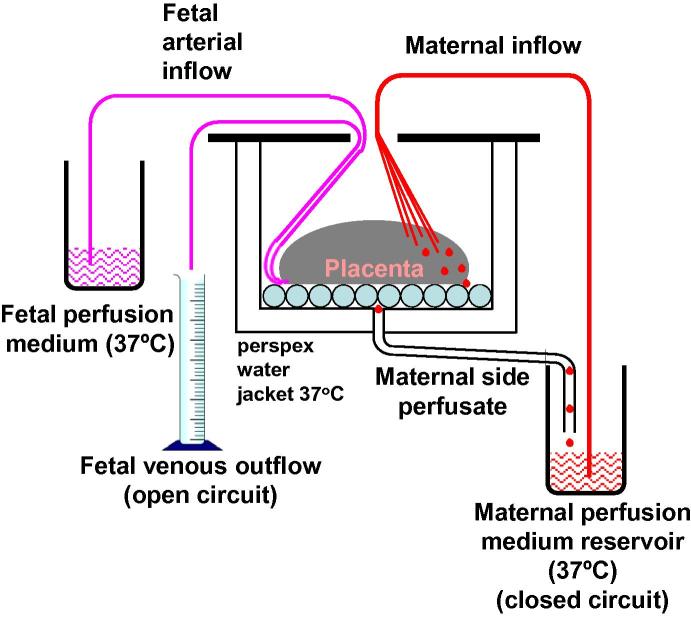
Schematic diagram of dual placental lobe perfusion system.

STBEV were prepared using a dual placental lobe perfusion system [18] modified as previously described [19], which involves the *ex vivo* reestablishment of the fetal and maternal circulations of an intact placental lobe. The fetal outflow is continuously measured to monitor the integrity of the lobes circulation and the maternal outflow is collected to harvest STBEV (Fig. 1) [2], [7], [20].

### 2.3 Maternal placental perfusate STBEV fractionation protocol

**Fig. 2:**
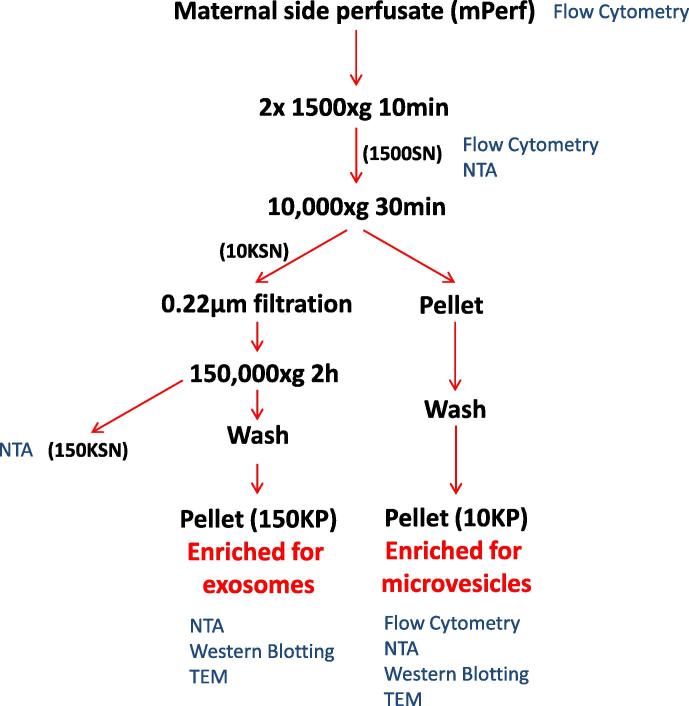
Schematic diagram of the sequential centrifugation and filtration protocol for the isolation and fractionation of placental EV from maternal perfusate collected using the *ex vivo* dual placental lobe perfusion method. SN = supernatant, NTA = Nanoparticle Tracking Analysis and TEM = transmission electron microscopy.

All processing was carried out on fresh maternal side perfusate (mPerf). At the end of the 3 h perfusion period, an aliquot of mPerf was set aside for flow cytometric analysis before centrifugation (Beckman Coulter Avanti J-20XP centrifuge using a Beckman Coulter JS-5.3 swing out rotor; 2 × 1500×*g* for 10 min at 4 °C) to remove contaminating RBC and large cellular debris (Fig. 2). The supernatant (1500SN) was collected and an aliquot again set aside for analysis, before centrifugation of the remaining 1500SN (Beckman L80 ultracentrifuge and Sorvall TST28.39 swing out rotor; 10,000×*g* for 30 min at 4 °C) (Fig. 2). The supernatant was removed and all pellets resuspended and pooled in sterile PBS before being centrifuged again (10,000×*g* for 30 min at 4 °C) to wash the pellet, further reducing contamination by soluble proteins. The resultant washed pellet (10KP) containing enriched microvesicles was then resuspended in sterile PBS for subsequent analysis (Fig. 2). The 10,000×*g* supernatant (10KSN) was passed through a 0.22 μm stericup filtration device (Millipore) before being centrifuged (150,000×*g* (maximum) for 2 h at 4 °C; Beckman L80 ultracentrifuge with a Sorvall TST28.39 swing out rotor) to pellet the remaining EV. The pellets were pooled and washed in sterile PBS before finally being resuspended in PBS to give an enriched exosome fraction (150KP) (Fig. 2). An aliquot of 150,000×*g* supernatant (150KSN) was kept for analysis. The protein content of each resuspended washed pellet was assessed using a BCA protein assay kit (Pierce) before aliquots were stored at −80 °C until subsequent use.

### 2.4 Flow cytometric analysis of mPerf, 1500SN and 10KP samples

A BD LSRII flow cytometer (BD Biosciences) was used to analyse the freshly isolated mPerf, 1500SN and 10KP samples. Multicolour flow cytometry with the pan EV membrane marker bio-maleimide (BODIPY FL N-(2-aminoethyl)-maleimide; Molecular Probes), STBEV marker PLAP and markers of potential contaminating platelet EV (CD41), RBC EV (CD235a/b) and all EV except those from STB and RBC (HLA class I) was carried out using the method previously described [4] (See Table 1 for antibody/dye details). Data were analysed using FACSDiva software version 8.0 (BD Biosciences) and figures generated using FlowJo version 10 (Tree Star Inc., Ashland, OR).

### 2.5 Nanoparticle Tracking Analysis

EV size distribution profiles and concentration measurements in fresh 1500SN, 10KP, 150KP and 150KSN were obtained using the NanoSight NS500 instrument equipped with a 405 nm laser (Malvern UK), sCMOS camera and Nanoparticle Tracking Analysis (NTA) software version 2.3, Build 0033 (Malvern UK). Silica 100 nm microspheres (Polysciences, Inc.) were routinely analysed to check instrument performance [21]. EV samples were diluted in PBS to give approximately 5 × 10^8^ EV/mL. The samples were automatically introduced into the sample chamber and the following script was used for EV measurements: PRIME, DELAY 5, CAPTURE 60, REPEAT 4. Samples were recorded using a camera level of 12 (Camera shutter speed; 15 ms and Camera gain; 350) and NTA post acquisition settings were optimised and kept constant between samples. Each video recording was analysed to give EV size and concentration measurements.

### 2.6 Transmission electron microscopy analysis of 10KP and 150KP samples

10KP and 150KP samples from one placental perfusion were fixed *in situ* with 4% glutaraldehyde in 0.1 M phosphate buffer. The pellets were then removed and processed for routine TEM. Samples were post-fixed in 2% osmium tetroxide in 0.1 M phosphate buffer, followed by dehydration in ethanol and treatment with propylene oxide prior to embedding in Spurr’s epoxy resin. Thin sections were cut and stained with uranyl acetate and lead citrate prior to examination using a Jeol 1200EX electron microscope.

### 2.7 Western blotting of 10KP and 150KP samples for markers of exosomes, STB origin and contaminating EV

Western blotting for the STB marker PLAP, exosomal markers Alix and CD63, and markers of potential contaminating EV CD41 (platelet EV marker), CD235a/b (RBC EV marker) and CD45 (leucocyte EV marker) was carried out using the method previously described [2]. Briefly, either 10 μg/well (PLAP, CD41, CD235a/b, CD45) or 25 μg/well (Alix and CD63) samples of total protein from lysed 10KP and 150KP samples and positive controls (RBC, platelets and leukocytes for CD235a/b, CD41 and CD45, respectively) were separated on 4–12% bis-tris gradient gels (Life Technologies) under reducing (all markers except CD63) or non-reducing (CD63) conditions. Separated proteins were then transferred onto PVDF membrane, nonspecific binding blocked with 5% blotto (Santa Cruz) and incubated with primary antibodies overnight at 4 °C (See Table 1 for details of antibodies). Membranes were then washed prior to incubation with an appropriate secondary antibody conjugated to horse radish peroxidase (Table 1). Membranes were washed again before incubation with an enhanced chemiluminescence substrate (Pierce) and exposure to Hyperfilm ECL (GE Health Care). All membranes were stained with Reactive Brown (0.5%; Sigma–Aldrich) to confirm equal protein loading (data not shown).

### 2.8 Fluorescence NTA

The existing fl-NTA technique for determining PLAP positive EV was improved by setting compatible scatter and fluorescence mode camera levels using ‘standard’ STBMV and STBEX samples containing known amounts of PLAP positive EV. STBMV and STBEX standards were prepared by determining the percentage of PLAP positive EV in pools of 10KP (STBMV standard) or 150KP (STBEX standard) (*n* = 4 individual preparations/pool, a subset of the 8 preparations used throughout this investigation), using immunodepletion with a saturating dose of anti-PLAP antibody coated Dynabeads. NTA was then used to determine the concentrations of the total EV [total] and unbound EV [PLAP negative]. The percentage of bound EV (PLAP positive) was calculated as: % PLAP positive = (([total] − [PLAP negative])/[total]) × 100. These pools were then labelled with quantum dots (Qdot) conjugated to anti-PLAP antibody (see Section 2.8.2). The NTA camera settings were then calibrated against the % PLAP positive measurements for the standards.

#### 2.8.1 Preparation of STB microvesicle and exosome standards

Pan mouse IgG Dynabeads (Life Technologies) were coated with either anti-PLAP antibody (NDOG2) or an isotype control (IgG1 Clone MOPC-21, Biolegend) according to the manufacturer’s instructions. The saturating concentration of anti-PLAP beads was determined by titrating anti-PLAP antibody or IgG1 beads against a fixed dose of 10KP or 150KP STBEV pool and using Western blotting for PLAP to show the bead concentration that removed all PLAP signal from the unbound EV portion. Briefly, STBEV (5 × 10^9^ EV/mL) were incubated with FcR blocking reagent (10 μl, 10 min at 4 °C), followed by overnight rotation (4 °C) alone or with increasing doses of anti-PLAP or IgG1 beads (10KP pool: 1 × 10^7^ to 4 × 10^7^ beads and 150KP pool: 2 × 10^6^ to 2 × 10^7^ beads) in a total volume of 1 mL. Bound and unbound EV were then separated and retained for PLAP Western blotting and NTA. For PLAP Western blotting, supernatant (20 μl) was mixed with 3× reducing buffer, while STBEV bound to beads were washed with PBS and solubilised in 1× reducing buffer before removal of beads by centrifugation (11,500×*g* for 1 min). An equivalent amount of the starting STBEV pools was also loaded onto the gels and PLAP Western blotting carried out as described above (Section 2.7). To determine the number of total and unbound EV, 10KP and 150KP pool samples and supernatants were diluted in PBS to approximately 5 × 10^8^ EV/mL and analysed using the NS500 instrument and NTA software as described above (Section 2.5).

#### 2.8.2 Fluorescence NTA: verification of settings and quantification of PLAP positive EV

Qdots were conjugated to anti-PLAP antibody NDOG2 or IgG1 isotype control antibody (IgG1 Clone MOPC-21, Biolegend) with a SiteClick Qdot 605 Antibody Conjugation Kit (Life Technologies) according to the manufacturer’s instructions, then filtered through Nanosep 0.2 μm centrifugal devices (Pall Life Sciences) prior to use. All Qdot conjugated antibodies were tested by flow cytometry to show that >90% of an STBEV sample labelled positive for PLAP and the conjugation was successful (data not shown). Next, the 10KP and 150KP standards, were diluted in PBS and incubated with FcR blocking reagent (10 μl, 10 min at 4 °C), followed by incubation with anti-PLAP-Qdot605 or IgG1-Qdot605 (10 μl, 1:100) for 20 min in the dark at room temperature. Samples were then topped up with PBS and analysed using the NanoSight NS500 instrument and NTA software. Samples were loaded into the chamber and the pump controlled to set a slow steady flow to minimise photo bleaching. The following samples were measured, firstly in scatter mode using a camera level of 12 (camera shutter speed 15 ms and camera gain 350), followed by fluorescence mode using a 430 nm long-pass filter and two camera levels, 12 and 13 (camera shutter speed 20 ms and camera gain 350) with 5 × 60 s videos captured for each sample, loading fresh sample into the chamber between each video capture: (1) STBEV alone, (2) STBEV + IgG1-Qdot605 and (3) STBEV + anti-PLAP-Qdot605 and background controls (4) FcR blocking reagent + IgG1-Qdot605 and (5) FcR blocking reagent + anti-PLAP-Qdot605. Using the scattered intensity vs. size plot, an exclusion box was set from 0–100 nm to exclude unbound single or aggregated Qdots from the analysis. NTA post acquisition settings were optimised and kept constant between samples measured under scatter mode and fluorescence mode. Background particles (∼0.2 × 10^8^–0.3 × 10^8^/mL) were subtracted from the 10KP and 150KP pool concentration and sizing data. Camera level 14 was also tested; however increased levels of background from unbound Qdots impeded the tracking of labelled EV (data not shown).

Once fl-NTA settings had been optimised, 10KP (*n* = 8) and 150KP (*n* = 8) samples were diluted in PBS and labelled as described above. Samples were measured in scatter mode using a camera level of 12 and in fluorescence mode using a camera level of 13. NTA post acquisition settings were kept constant between samples measured under scatter and fluorescence modes.

## 3 Results and discussion

### 3.1 Flow cytometric analysis of mPerf, 1500SN and 10KP samples

**Fig. 3:**
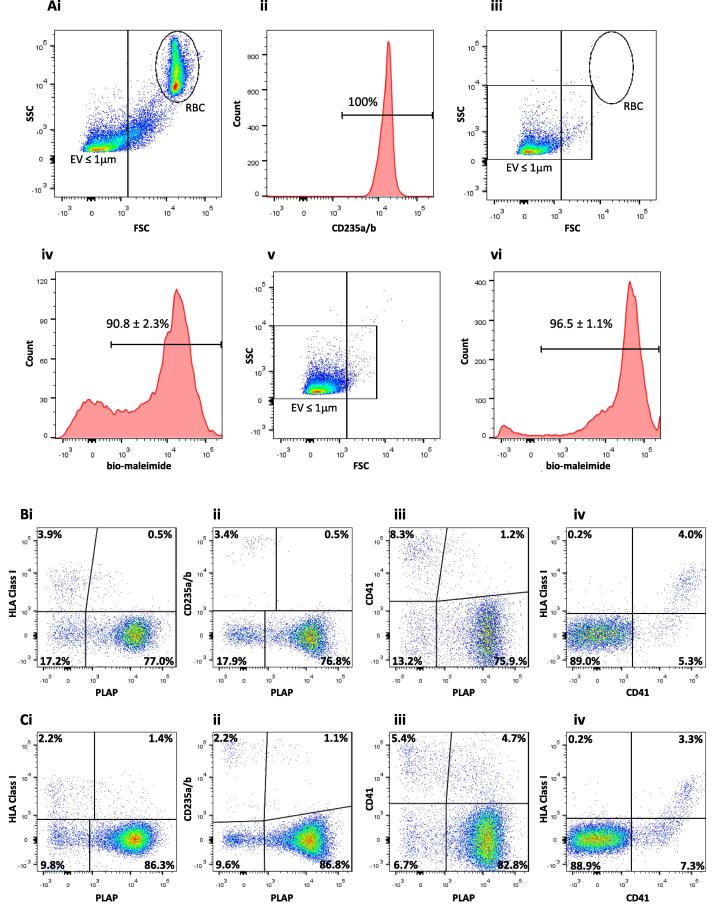
Representative flow cytometric analyses of fractionated maternal side placental perfusate. (Ai) Forward scatter (FSC) vs. side scatter (SSC) profile of maternal side placental perfusate, (Aii) histogram showing 100% labelling of red blood cells (RBC), (Aiii) maternal side placental perfusate supernatant (1500SN) following depletion of contaminating RBC by 2 × 1500×*g* centrifugation for 10 min, (Aiv) 1500SN bio-maleimide positive labelling, (Av) FSC vs. SSC profile of 10,000×*g* pellet (10KP) and (Avi) 10KP bio-maleimide positive labelling. (Bi) 1500SN bio-maleimide positive EV displayed on two-parameter plots and phenotype identified: syncytiotrophoblast derived EV (STBEV), PLAP^+^/HLA Class I^−^, (Bii) RBC EV, CD235a/b^+^/PLAP^−^, (Biii) platelet EV (CD41^+^/PLAP^−^), (Biv) leukocyte/endothelial EV (HLA Class I^+^/CD41^−^). (Ci) 10KP bio-maleimide positive EV displayed on two-parameter plots and phenotype identified: STBEV, PLAP^+^/HLA Class I^−^, (Cii) RBC EV, CD235a/b^+^/PLAP^−^, (Ciii) platelet EV (CD41^+^/PLAP), (Civ) leukocyte/endothelial EV (HLA Class I^+^/CD41^−^). Histograms show the mean ± SE for percentage positive EV and two-parameter plots show the mean for percentage positive EV (*n* = 8). Percentages have been adjusted to account for background contaminating particulates.

Despite a 20 min equilibration period, during which perfusates from the maternal side were discarded, freshly collected mPerf (*n* = 8) contained high numbers of contaminating RBC (54.9 ± 4.9%), visible as a discrete CD235a/b positive population, clearly shown by displaying events on a side scatter (SSC) vs. forward scatter (FSC) plot (Fig. 3Ai and Aii). Centrifugation at 1500×*g* (2 × 10 min) effectively and consistently removed the contaminating RBC population from the mPerf, together with most of the EV above 1 μm in size, with <1.0% RBC left in the 1500SN (Fig. 3Aiii). Five colour flow cytometric analysis showed that 90.8 ± 2.3% of the 1500SN EV population labelled positive with the cell membrane dye bio-maleimide (Fig. 3Aiv) and these EV were displayed on two-parameter dot plots showing that the 1500SN contained predominantly STB derived EV, (PLAP^+^/HLA Class I^−^: 77.0 ± 3.0%; Fig. 3Bi) and very low levels of contamination by RBC EV (CD235a/b^+^/PLAP^−^: 3.4 ± 0.9%; Fig. 3Bii), platelet EV (CD41^+^/PLAP^−^: 8.3 ± 2.0%; Fig. 3Biii) and leukocyte/endothelial EV (HLA Class I^+^/CD41^−^: 0.2 ± 0.1%; Fig. 3Biv). Flow cytometric analysis of the 10KP showed that 96.9 ± 0.2% of the population were <1 μm in size (Fig. 3Av) and 96.5 ± 1.1% labelled with bio-maleimide (Fig. 3Avi). Two-parameter dot plots displaying bio-maleimide positive 10KP EV showed further enrichment for those derived from STB (PLAP^+^/HLA Class I^−^: 86.3 ± 2.8%; Fig. 3Ci) and again very low levels of contaminating RBC EV (CD235a/b^+^/PLAP^−^: 2.2 ± 0.5%; Fig. 3Cii), platelet EV (CD41^+^/PLAP^−^: 5.4 ± 1.7%; Fig. 3Ciii) and leukocyte/endothelial EV (HLA Class I^+^/CD41^−^: 0.2 ± 0.1%; Fig. 3Civ). Hence the dual placental lobe perfusion model yields a population of 10KP STBEV that are highly enriched for PLAP with very low contamination by platelet, RBC, leukocyte and endothelial EV. Our data (1500SN and 10KP) also show a small population of PLAP^+^/HLA Class I^+^ EV (1500SN: 0.5 ± 0.1%; Fig. 3Bi and 10KP: 1.4 ± 0.2% Fig. 3Ci) and PLAP^+^/CD41^+^ EV (1500SN: 1.2 ± 0.2%; Fig. 3Biii and 10KP: 4.7 ± 0.8%; Fig. 3Ciii) suggesting that STBEV and platelet EV aggregate to a limited degree during sample processing. A small population of STBEV also appear to aggregate with RBC EV in the 1500SN and 10KP as shown by PLAP^+^/CD235a/b^+^ EV (0.5 ± 0.1%; Fig. 3Bii and 1.1 ± 0.4%; Fig. 3Cii, respectively). If ultrapure preparations are required, any contaminating EV that remain in the 1500SN could be removed by immunobead depletion prior to high speed centrifugation (data not shown). The 150KP was diluted to the same concentration as the 10KP, however at this concentration only a small number of EV were detected, as the majority were too small to be analysed using this multicolour flow cytometry technique (data not shown).

### 3.2 Nanoparticle Tracking Analysis and transmission electron microscopy

**Fig. 4:**
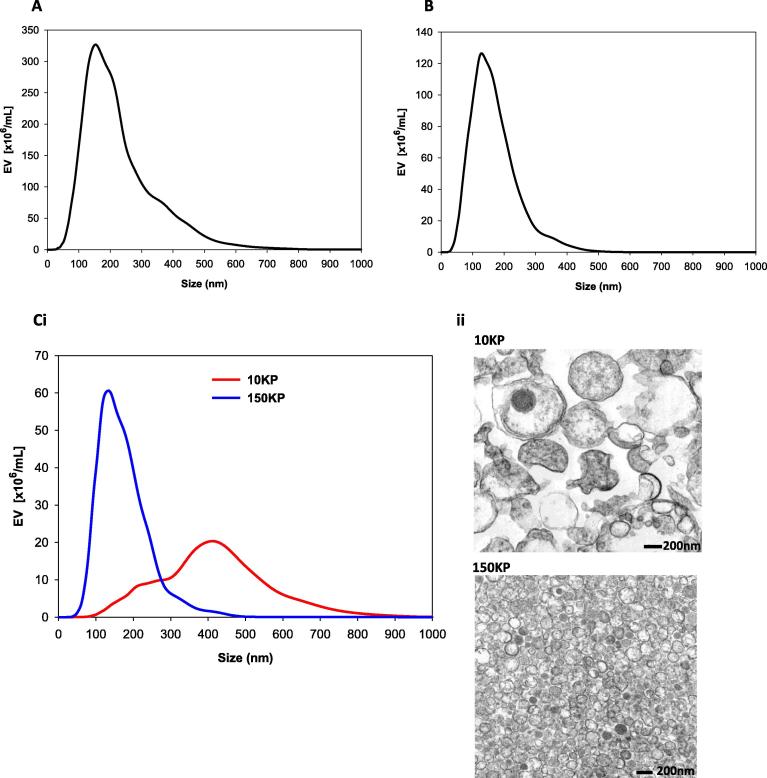
Nanoparticle Tracking Analysis size distribution profiles (mean data *n* = 8) for maternal side placental perfusate derived samples collected at the following stages of the fractionation process. (A) 2 × 1500×*g* supernatant (1500SN), (B) 150,000×*g* supernatant (150KSN), (Ci) 10,000×*g* pellet (10KP) and 150,000×*g* pellet (150KP). (Cii) Transmission electron micrographs of 10KP and 150KP. Scale bars represent 200 nm.

The lack of sensitivity of conventional flow cytometry for EV detection has led to the adoption of NTA for this purpose. Freshly collected mPerf could not be analysed using NTA owing to the high number of contaminating RBC. After they were removed by centrifugation at 1500×*g* (2 × 10 min) the supernatant (1500SN) contained diverse EV ranging in size from 50–800 nm in diameter, with an average modal size of 159 ± 8.0 nm (Fig. 4A). Individual 10KP samples (1–8) contained EV with an average modal size of 395 ± 12 nm, suggesting they are predominantly microvesicles (Table 2 and Fig. 4Ci). The final 0.22 μm filtration and 150,000×*g* centrifugation step (2 h) of the 10KSN yielded a 150KP with EV of a 147 ± 6 nm average modal size and 50–400 nm size range (outlined in Table 2), consistent with enrichment for exosomes (Fig. 4Ci). TEM confirmed the NTA data: showing that the 10KP was enriched for EV >300 nm which were heterogeneous in size and appearance (Fig. 4Cii), while the 150KP contained a more homogeneous population of small EV (approximately 50–200 nm), in the reported size range of exosomes (Fig. 4Cii).

The 150KSN contained EV or particles that could not be pelleted (50–450 nm), with an average modal size of 134 ± 6 nm (Fig. 4B). Preliminary analysis suggests that these are lipoprotein contaminants that predominantly remain after centrifugation at 150,000×*g*. This suggests that ultracentrifugation separates exosomes effectively from more buoyant lipoprotein moieties which are unavoidable when samples are or may be contaminated with blood.

### 3.3 Western blotting of 10KP and 150KP samples for markers of exosomes, STB origin and contaminating EV

**Fig. 5:**
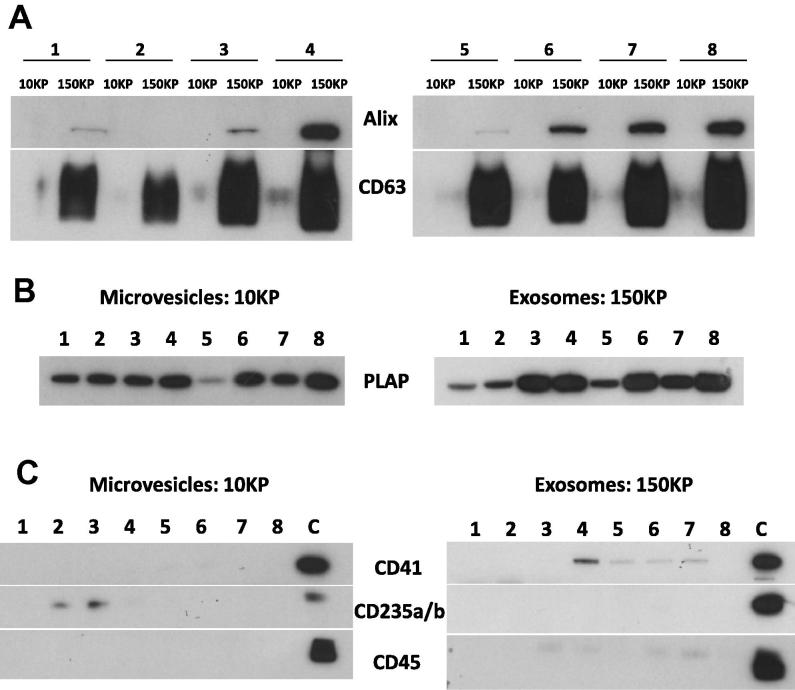
Representative immunoblot images of the following EV markers: (A) Alix and CD63 (exosomes), (B) Placental alkaline phosphatase (PLAP; syncytiotrophoblast (STB)), (C) CD41 (platelet), CD235a/b (red blood cell) and CD45 (leucocyte) in 10,000×*g* (10KP) and 150,000×*g* (150KP) fraction samples. Samples 1–8 are individual STBEV preparations and sample C is a positive control for each marker of interest.

It is not possible to analyse EV in the 150KP and also some of those in the 10KP using our LSRII flow cytometer, as these are below the instruments lower limit of detection. Therefore, in addition to multicolour flow cytometry, 10KP and 150KP samples (1–8) were analysed using Western blotting. Analysis of the 10KP and 150KP samples showed enrichment of the exosome markers Alix and CD63 in the 150KP samples, with virtually none in the 10KP, which reinforces our evidence (above) that our method enriches exosomes in the 150KP fraction of placental perfusate EV (Fig. 5A). All 10KP and 150KP samples were PLAP positive, consistent with their origin from STB (Fig. 5B). Western blotting analysis of these preparations for markers of platelets, RBC and leucocytes showed either small bands or no signal compared to the positive controls, suggesting very low amounts of contaminating EV (Fig. 5C).

We clearly show that all 10KP and 150KP samples (1–8) express PLAP, however Western blot analysis is only semi-quantitative. Next, we aimed to quantify PLAP positive EV in 10KP and 150KP samples using fl-NTA.

### 3.4 Fluorescence NTA

#### 3.4.1 Preparation of STB microvesicle and exosome standards

**Fig. 6:**
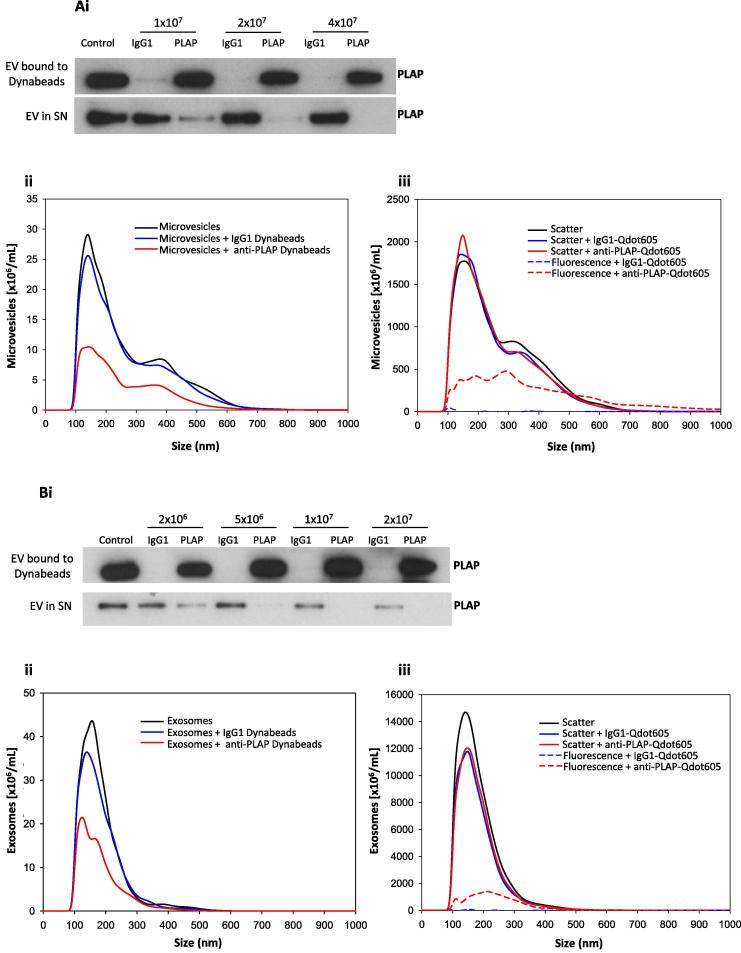
Immunobead depletion and Fluorescence-NTA (fl-NTA) measurements (*n* = 3 replicates) of 10,000×*g* (10KP) and 150,000×*g* (150KP) fraction pools. (Ai) Representative immunoblot image of placental alkaline phosphatase (PLAP) in the 10KP pool showing bound EV (PLAP positive) or those remaining in the supernatant (SN; PLAP negative) with increasing doses of IgG1 Dynabeads or anti-PLAP Dynabeads. (Aii) NTA of 10KP pool alone (black line) or post incubation with a saturating concentration [4 × 10^7^] of IgG1 Dynabeads (blue line) or anti-PLAP Dynabeads (red line). (Aiii) fl-NTA of the 10KP pool measured in; scatter mode: alone (black line), labelled with IgG1-Qdot605 control (blue solid line) or labelled with anti-PLAP-Qdot605 antibody (red solid line) and fluorescence mode: labelled with IgG1-Qdot605 control (blue dash) or anti-PLAP-Qdot605 antibody (red dash). (Bi) Representative immunoblot image of PLAP in the 150KP pool showing bound EV (PLAP positive) or those remaining in the supernatant (SN; PLAP negative) in response to an increasing dose (0.2–2 × 10^7^) of IgG1 Dynabeads or anti-PLAP Dynabeads. (Bii) NTA of 150KP pool alone (black line) or post incubation with a saturating concentration [1 × 10^7^] of IgG1 Dynabeads (blue line) or anti-PLAP Dynabeads (red line). (Biii) Fl-NTA of the 150KP pool measured in; scatter mode: alone (black line), labelled with IgG1-Qdot605 control (blue solid line) or labelled with anti-PLAP-Qdot605 antibody (red solid line) and fluorescence mode: labelled with IgG1-Qdot605 control (blue dash) or anti-PLAP-Qdot605 antibody (red dash).

To prepare a STBEV ‘standard’, the saturating doses of anti-PLAP beads were first determined for 10KP and 150KP pools (prepared from freeze/thawed samples). 4 × 10^7^ and 1 × 10^7^ of anti-PLAP beads were effective at removing all PLAP signal from 1 mL of the 10KP and 150KP pools respectively at 5 × 10^9^ EV/mL and were chosen as the saturating doses in subsequent analyses (Fig. 6Ai and Bi). NTA of the supernatant from 10KP pool incubated alone or with IgG1 coated beads showed similar concentrations (5.32 × 10^9^ EV/mL and 4.85 × 10^9^ EV/mL, respectively) (Fig. 6Aii), indicating very low levels of nonspecific binding. Following incubation of 10KP with anti-PLAP beads, 59.8 ± 5.2% of the EV were depleted and therefore STB derived (Fig. 6Aii). Similar NTA data was obtained for the 150KP pool. Comparable concentration measurements for the 150KP pool supernatant after incubation alone or with IgG1 coated beads (5.01 × 10^9^ EV/mL and 4.48 × 10^9^ EV/mL respectively) showed minimal levels of non-specific binding (Fig. 6Bii). Incubation of 150KP pool with anti-PLAP beads showed that PLAP positive STBEV constituted 51.6 ± 3.4% of the 150KP pool (Fig. 6Bii).

#### 3.4.2 Quantification of PLAP positive EV using fluorescence NTA

Analysis of the 10KP and 150KP pools alone and labelled with IgG1-Qdot605 or anti-PLAP-Qdot605 antibody, in scatter mode showed overlapping size profiles (10KP pool modal size: 160 ± 3, 162 ± 14 and 153 ± 8 nm and 150KP pool modal size: 143 ± 1, 149 ± 3 and 148 ± 2 nm, respectively; Fig. 6Aiii and Biii) and concentration measurements (10KP pool: 3.95 × 10^11^, 3.73 × 10^11^ and 3.93 × 10^11^ EV/mL and 150KP pool: 18.3 × 10^11^, 14.7 × 10^11^ and 14.9 × 10^11^ EV/mL, respectively), showing that bound Qdots did not affect EV physical characteristics. Analysis of these same 10KP and 150KP pools using fluorescence mode showed no measurable autofluorescence (data not shown) and very low levels of background fluorescence with IgG1-Qdot605 at camera levels 12 and 13 (10KP pool: 1.4 ± 0.5% and 0.4 ± 0.3% and 150KP pool: 0.2 ± 0.2% and 0.5 ± 0.5%, respectively; Fig. 6Aiii and Biii). Analysis of anti-PLAP-Qdot605 labelled pools showed that camera level 13 gave the highest values (10KP pool 39.8 ± 3.0% and 48.5 ± 3.0% and 150KP: 15.0 ± 1.9% and 17.9 ± 3.3% at camera levels 12 and 13, respectively) and, in the case of 10KP pool, most comparable to that obtained with anti-PLAP beads (10KP pool: 59.8 ± 5.2% and 150KP pool: 51.6 ± 3.4%), suggesting that camera level 13 was the best setting for detecting PLAP positive STBEV in fluorescence mode (Fig. 6Aiii and Biii). Therefore, individual 10KP (*n* = 8) and 150KP (*n* = 8) samples (freeze/thawed) were analysed using scatter mode camera level 12 and fluorescence mode camera level 13. The size distribution profile of the 10KP pool labelled with the anti-PLAP-Qdot605 antibody and measured under fluorescence mode showed noticeably larger EV when compared to analysis in scatter mode (Fig. 6Aiii). This is due to the fluorescence measurement increasing in brightness approximately proportional to radius^2^ (surface area of a sphere), whereas NTA scatter measurements increase in brightness proportional to up to *r*^6^, making large EV harder to accurately track in scatter mode, as they saturate the camera sensor chip.

The fl-NTA results of individual 10KP and 150KP samples are shown in Table 2. The average modal size of EV measured in scatter mode was 157 ± 12 nm and 142 ± 4 nm in the 10KP and 150KP samples, respectively. The fl-NTA sizing data of the freeze/thawed 10KP samples clearly show that the average modal and mean sizes have significantly decreased (*p* < 0.001 Student *t*-test) in comparison to analysis of the same individual samples when fresh (Section 3.2, Table 2). This shows that STBMV are affected by freezing, perhaps due to shrinking or fragmentation into smaller EV. Freezing did not appear to affect the 150KP pool sample in the same way, as sizing measurements were similar for both freeze/thawed and fresh samples (Table 2). Using camera 13, low background fluorescence was measured with IgG1-Qdot605 labelled 10KP and 150KP samples (range: 0.0–2.4%). The percentage of PLAP positive EV varied amongst individual preparations, ranging from 17.8–66.9% in the 10KP samples and 3.3–51.7% in the 150KP samples (Table 2). The number of PLAP positive EV in all samples was sufficient to enable accurate sizing and concentration measurement, with the exception of sample 5 (10KP and 150KP) whereby the data is less reliable due to the low concentration of EV (<2 × 10^8^/mL) [21]. It is noteworthy that individual 10KP and 150KP samples 2–5 were those used in the 10KP and 150KP pools. The average percentage of PLAP positive EV for 10KP and 150KP samples 2–5 showed good consistency with results for the pooled samples (10KP: 49.0 ± 10.7% vs. 48.5 ± 3.0% and 150KP: 23.1 ± 7.5% vs. 17.9 ± 3.3%, respectively).

Our data shows that the use of anti-PLAP immunobead depletion has provided the necessary standard to optimise fl-NTA camera settings. The percentage of PLAP positive EV in the 10KP pool analysed by fl-NTA was comparable to that using anti-PLAP beads and shows that fl-NTA is a robust method for analysing PLAP on STBMV. However, using fl-NTA to quantify PLAP in the 150KP pool, detected fewer PLAP positive EV in comparison to using anti-PLAP beads, showing that fl-NTA is not as sensitive as immunobead capture for the detection of PLAP on STBEX. This may be due to the smaller STBEX having less surface PLAP. Reduction of background fluorescence by removal of unbound Qdots may also enable a higher camera level to be used, increasing instrument sensitivity. This may also be improved when analysing a marker enriched on the surface of exosomes i.e. CD63. Furthermore, the antigen labelling methods available for the detection of microvesicles and exosomes are still limited. The development of novel probes may also help to improve the labelling of exosomes for increased detection using fl-NTA.

Anti-PLAP immunobead depletion and fl-NTA show that not all EV present in STBMV and STBEX preparations are PLAP positive. However, this does not necessarily indicate they are derived from another source and not from the STB layer. Flow cytometry and western blot analyses demonstrated enrichment of STBEV and low levels of contaminating blood derived EV, suggesting that PLAP may only be sorted into some STBEV or the amount of PLAP antigen on the EV surface is too low for immunodetection with our anti-PLAP antibody (NDOG2), used for both anti-PLAP immunobead depletion and fl-NTA. Alternative STBEV specific markers that may be used to identify STBMV and STBEX are currently under investigation.

## 4 Conclusions

Pregnancy is a unique situation in EV research as the whole organ (the placenta) which is the source of the EV of interest becomes available for study after the baby is delivered. This is particularly relevant for pre-eclampsia which together with some other pregnancy specific disorders is placentally mediated. In pre-eclampsia STBEV release is altered, fuelling interest in their potential as biomarkers of placental dysfunction and therapeutic targets. We have shown that *ex vivo* derived maternal side placental perfusate is a rich source of STBEV and have developed a robust method of isolating enriched preparations of STBMV and STBEX, with low levels of contaminating platelet, RBC and leucocyte EV. This is also a rare example of *ex vivo* EV preparation from an intact organ. We have also determined appropriate scatter and fluorescence mode fl-NTA settings to use for STBEV labelled with anti-PLAP antibody conjugated to Qdots and demonstrated that fl-NTA can be used to measure the proportion of PLAP positive EV in preparations of both microvesicles and exosomes. These developments will significantly aid our progress to better understand the role played by STBEV in normal and pathological pregnancies.

## Figures and Tables

**Table 1:** Fluorescent label, antibodies, isotype controls and secondary antibody used for flow cytometry and Western blot analysis.

Antibody/dye	Concentration or dilution	Antigen	EV specificity	Source
*Flow cytometry*				
Bio-maleimide (BODIPY FL *N*-(2-aminoethyl)-maleimide)	0.5 μM	Thiol groups	All EV	Molecular probes
aAnti PLAP-PE (NDOG2)	0.5 μg/ml	PLAP	STBEV	In-house antibody
IgG1-PE (clone MOPC-21)	0.5 μg/ml	Isotype control		Biolegend
Anti HLA class I-Alexa 647 (clone W6/32)	2.5 μg/ml	HLA class I	All EV except STB and RBC derived	AbD Serotec
IgG2a-Alexa 647 (clone MRC OX-34)	2.5 μg/ml	Isotype control		AbD Serotec
Anti CD41-PECy7 (clone P2)	0.25 μg/ml	CD41	Platelet EV	Beckman Coulter
IgG1-PECy7 (clone MOPC-21)	0.25 μg/ml	Isotype control		Beckman Coulter
Anti CD235a/b-PECy5 (clone HIR2)	0.05 μg/ml	CD235a/b	RBC EV	Biolegend
IgG2b-PECy5 (clone MPC-11)	0.05 μg/ml	Isotype control		Biolegend

*Western blotting*				
Anti PLAP (NDOG2)	1 μg/ml	PLAP	STBEV	In-house antibody
Anti Alix (clone 3A9)	1/1000	Alix	Exosomes	Cell signaling technology
Anti CD63 (clone MEM-259)	1 μg/ml	CD63	Exosomes	Abcam
Anti CD41 (clone 745201)	2 μg/ml	CD41	Platelet EV	R&D systems
Anti CD235a/b (clone HIR2)	0.5 μg/ml	CD235a/b	RBC EV	Santa-Cruz
Anti CD45 (clones 2BII + PD7/26)	0.375 μg/ml	CD45	Leucocyte EV	Dako
Polyclonal goat anti-mouse immunoglobulin HRP	0.25 μg/ml	Mouse immunoglobulins		Dako

a In-house antibody, custom conjugation by Biolegend.

**Table 2:** Results of NTA and fluorescence NTA of individual 10KP and 150KP samples (1–8) showing mean and mode EV size (nm) and positivity with IgG1 control (%) and anti-PLAP Qdot labelling (%).

Sample	Fresh: SCATTER		Freeze/thawed: SCATTER		Freeze/thawed: FLUORESCENCE PLAP POSITIVE		IgG1-Qdot605	PLAP-Qdot605
Mean EV size (nm)	Mode EV size (nm)	Mean EV size (nm)	Mode EV size (nm)	Mean EV size (nm)	Mode EV size (nm)	Positive (%)	Positive (%)
*10KP*								
1	440 ± 12	440 ± 16	306 ± 11	217 ± 42	527 ± 27	372 ± 28	0.0	56.8
2	420 ± 12	410 ± 18	295 ± 12	160 ± 5	520 ± 11	312 ± 22	0.0	66.2
3	403 ± 33	389 ± 37	300 ± 9	157 ± 11	541 ± 11	332 ± 26	0.0	58.5
4	397 ± 15	385 ± 44	281 ± 11	193 ± 15	436 ± 22	331 ± 26	0.1	53.6
5	370 ± 14	338 ± 36	226 ± 14	137 ± 4	607 ± 77	126 ± 14	1.1	17.8
6	375 ± 12	362 ± 44	242 ± 22	140 ± 8	525 ± 18	241 ± 19	2.0	65.7
7	431 ± 26	403 ± 60	234 ± 9	124 ± 4	581 ± 20	239 ± 59	2.4	50.4
8	429 ± 19	432 ± 30	247 ± 12	127 ± 10	561 ± 16	229 ± 30	0.9	66.9
Average	408 ± 9	395 ± 12	266 ± 11⁎	157 ± 12⁎				54.5 ± 5.7

*150KP*								
1	179 ± 5	163 ± 14	164 ± 2	129 ± 6	205 ± 4	159 ± 7	0.0	23.4
2	172 ± 2	158 ± 7	183 ± 3	141 ± 7	238 ± 6	203 ± 18	0.0	34.0
3	185 ± 2	149 ± 5	195 ± 3	161 ± 8	239 ± 6	160 ± 18	0.0	35.4
4	182 ± 6	135 ± 3	168 ± 1	140 ± 4	199 ± 3	153 ± 18	0.0	19.7
5	159 ± 2	118 ± 1	176 ± 2	138 ± 3	378 ± 99	170 ± 60	2.2	3.3
6	185 ± 7	158 ± 11	201 ± 6	144 ± 6	375 ± 23	163 ± 17	0.1	49.0
7	179 ± 9	150 ± 12	179 ± 2	149 ± 6	231 ± 7	148 ± 13	0.0	28.1
8	185 ± 1	145 ± 9	198 ± 4	131 ± 6	256 ± 10	163 ± 17	0.0	51.7
Average	178 ± 3	147 ± 6	183 ± 5	142 ± 4				30.6 ± 5.6

Data show mean ± SE of five individual video recordings.

⁎ *P *< 0.001 10KP fresh scatter mean and mode EV size (nm) vs. 10KP freeze/thawed scatter mean and mode EV size (nm).
